# Patterns in Use and Transplant Outcomes Among Adult Recipients of Kidneys From Deceased Donors With COVID-19

**DOI:** 10.1001/jamanetworkopen.2023.15908

**Published:** 2023-05-30

**Authors:** Mengmeng Ji, Amanda J. Vinson, Su-Hsin Chang, Massini Merzkani, Krista L. Lentine, Yasar Caliskan, Kristin Progar, Nicole Nesselhauf, Casey Dubrawka, Tarek Alhamad

**Affiliations:** 1 Division of Nephrology, Washington University in St Louis, St Louis, Missouri; 2 Division of Public Health Sciences, Washington University in St Louis, St Louis, Missouri; 3 Division of Nephrology, Department of Medicine, Dalhousie University, Halifax, Nova Scotia, Canada; 4 St Louis University Transplant Center, SSM Health St Louis University Hospital, St Louis, Missouri; 5 Department of Pharmacy, Barnes-Jewish Hospital, St Louis, Missouri

## Abstract

**Importance:**

While the COVID-19 pandemic enters a new phase and the proportion of individuals with a previous COVID-19 diagnosis increases, the national patterns in kidney use and medium-term kidney transplant (KT) outcomes among patients receiving kidneys from active or resolved COVID-19–positive donors remain unknown.

**Objective:**

To evaluate the patterns in kidney use and KT outcomes among adult recipients of kidneys from deceased donors with active or resolved COVID-19.

**Design, Setting, and Participants:**

This retrospective cohort study was conducted using national US transplant registry data from 35 851 deceased donors (71 334 kidneys) and 45 912 adult patients who received KTs from March 1, 2020, to March 30, 2023.

**Exposure:**

The exposure was donor SARS-CoV-2 nucleic acid amplification test (NAT) results, with positive NAT results within 7 days before procurement defined as active COVID-19 and positive NAT results 1 week (>7 days) before procurement defined as resolved COVID-19.

**Main Outcomes and Measures:**

Primary outcomes were kidney nonuse, all-cause kidney graft failure, and all-cause patient death. Secondary outcomes were acute rejection (ie, rejection in the first 6 months after KT), transplant hospitalization length of stay (LOS), and delayed graft function (DGF). Multivariable logistic regression analyses were performed for kidney nonuse, rejection, and DGF; multivariable linear regression analyses were performed for LOS; and multivariable Cox regression analyses were performed for graft failure and all-cause death. All models were adjusted for inverse probability treatment weighting.

**Results:**

Among 35 851 deceased donors, the mean (SD) age was 42.5 (15.3) years; 22 319 (62.3%) were men and 23 992 (66.9%) were White. Among 45 912 recipients, the mean (SD) age was 54.3 (13.2) years; 27 952 (60.9%) were men and 15 349 (33.4%) were Black. The likelihood of nonuse of kidneys from active or resolved COVID-19–positive donors decreased over time. Overall, kidneys from active COVID-19–positive donors (adjusted odds ratio [AOR], 1.55; 95% CI, 1.38-1.76) and kidneys from resolved COVID-19–positive donors (AOR, 1.31; 95% CI, 1.16-1.48) had a higher likelihood of nonuse compared with kidneys from COVID-19–negative donors. From 2020 to 2022, kidneys from active COVID-19–positive donors (2020: AOR, 11.26 [95% CI, 2.29-55.38]; 2021: AOR, 2.09 [95% CI, 1.58-2.79]; 2022: AOR, 1.47 [95% CI, 1.28-1.70]) had a higher likelihood of nonuse compared with kidneys from donors without COVID-19. Kidneys from resolved COVID-19–positive donors had a higher likelihood of nonuse in 2020 (AOR, 3.87; 95% CI, 1.26-11.90) and 2021 (AOR, 1.94; 95% CI, 1.54-2.45) but not in 2022 (AOR, 1.09; 95% CI, 0.94-1.28). In 2023, kidneys from both active COVID-19–positive donors (AOR, 1.07; 95% CI, 0.75-1.63) and resolved COVID-19–positive donors (AOR, 1.18; 95% CI, 0.80-1.73) were not associated with higher odds of nonuse. No higher risk of graft failure or death was found in patients receiving kidneys from active COVID-19–positive donors (graft failure: adjusted hazard ratio [AHR], 1.03 [95% CI, 0.78-1.37]; patient death: AHR, 1.17 [95% CI, 0.84-1.66]) or resolved COVID-19–positive donors (graft failure: AHR, 1.10 [95% CI, 0.88-1.39]; patient death: AHR, 0.95 [95% CI, 0.70-1.28]). Donor COVID-19 positivity was not associated with longer LOS, higher risk of acute rejection, or higher risk of DGF.

**Conclusions and Relevance:**

In this cohort study, the likelihood of nonuse of kidneys from COVID-19–positive donors decreased over time, and donor COVID-19 positivity was not associated with worse KT outcomes within 2 years after transplant. These findings suggest that the use of kidneys from donors with active or resolved COVID-19 is safe in the medium term; further research is needed to assess longer-term transplant outcomes.

## Key Points

**Question:**

What are the national patterns in kidney use and transplant outcomes among adult recipients of kidneys from deceased donors with active or resolved COVID-19?

**Findings:**

In this cohort study of 35 851 deceased donors and 45 912 adult transplant recipients in the US, the likelihood of nonuse of kidneys from active or resolved COVID-19–positive donors decreased over time, with no higher likelihood of nonuse for kidneys from COVID-19–positive donors in 2023. Donor COVID-19 positivity was not associated with worse transplant outcomes within 2 years after transplant.

**Meaning:**

These findings suggest that the use of kidneys from donors with active or resolved COVID-19 is safe, with excellent outcomes during medium-term follow-up.

## Teaser

This cohort study uses national US transplant registry data to assess patterns in kidney use and kidney transplant outcomes among adult recipients of kidneys from deceased donors with active or resolved COVID-19.

## Introduction

The COVID-19 pandemic caused an immediate and substantial decrease in solid organ transplant rates globally,^1,2,3^ with kidney transplants (KTs) most affected, followed by lung, liver, and heart transplants.^1,4^ In the US, KT is the most common type of solid organ transplant, with a record 26 228 KTs performed in 2022.^5,6^ The pandemic has required KT professionals (including transplant surgeons and nephrologists) to make difficult decisions about the use of kidneys from donors with SARS-CoV-2 infection, for which the potential risks of infectivity had to be weighed against the discard of valuable organs.^4^

At the beginning of the pandemic, COVID-19–positive patients were not considered eligible to be potential living or deceased donors, resulting in decreased transplant volume.^7,8^ From August 8, 2020, to September 29, 2021, only 150 SARS-CoV-2 nucleic acid amplification test (NAT)–positive deceased donors were assessed for organ donation.^9^ Among 295 procured kidneys from SARS-CoV-2 NAT-positive donors, 102 (34.6%) were not used compared with a nonuse rate of 20.6% for kidneys from NAT-negative donors.^9^ This high nonuse rate of kidneys from SARS-CoV-2 NAT-positive donors was cited as primarily an absence of recipient availability^9,10^; however, the uncertainty regarding potential SARS-CoV-2 transmission risk and the relative risk-benefit profile of such organs likely also influenced decision-making.

Previous studies have reported decreasing patterns of kidney use and KT practices during the pandemic^2,9,10^; however, these analyses were mainly descriptive and thus did not control for detailed donor characteristics. In addition, to our knowledge, no study has investigated the use of kidneys from COVID-19–positive donors as the pandemic enters a new phase in 2023, which comprises evolving SARS-CoV-2 variant strains, increased understanding of COVID-19 risks and treatment strategies, availability of COVID-19 vaccines, and accrual of a small body of evidence suggesting that at least short-term outcomes are generally acceptable for the transplanting of nonlung COVID-19–positive donor organs.^11^

Importantly, as the proportion of individuals with a previous COVID-19 diagnosis increases, the impact of previous COVID-19 diagnosis in potential kidney donors also warrants consideration. A recent autopsy study^12^ found persistent SARS-CoV-2 organ and brain infection in patients with a COVID-19 diagnosis as early as 230 days before death. Therefore, it is possible that even resolved COVID-19 in donors may be associated with risk at the time of organ donation. Similarly, whether the nonuse of kidneys from donors with previous resolved COVID-19 is higher also requires consideration. In the context of these ongoing important questions, we conducted a study to explore the patterns in nonuse of kidneys from deceased donors with active or previous COVID-19 and KT outcomes among adult recipients using 2020 to 2023 national registry data, controlling for a comprehensive list of donor and recipient characteristics.

## Methods

Exemptions for study approval and informed consent were obtained for this cohort study from the Washington University in St Louis Institutional Review Board because the study involved only secondary analyses of deidentified data. This study followed the Strengthening the Reporting of Observational Studies in Epidemiology (STROBE) reporting guideline for cohort studies.

### Study Population and Design

The Organ Procurement and Transplantation Network (OPTN) database contains data on all transplant waiting list candidates, recipients, and donors in the US, including details about their demographic and clinical characteristics at the time of waiting list entry and transplant.^13,14,15^ We limited the analysis to deceased donors and KT recipients after March 1, 2020, when the World Health Organization declared COVID-19 a pandemic. Exclusion criteria for recipients included (1) younger than 18 years at the time of transplant, (2) multiorgan transplant (eg, pancreas-kidney transplant), and (3) previous KT. These data were obtained from the March 2023 version of the OPTN Standard Analysis and Research File.^15^

### Donor COVID-19 Status

The exposure of interest was donor SARS-CoV-2 NAT results and antibody test results. Days between test specimen collection date and kidney recovery date were calculated. A positive SARS-CoV-2 NAT result within 7 days before procurement was defined as an active COVID-19 infection (COVID-19 positive). Donors who ever had a positive upper or lower SARS-CoV-2 NAT result 1 week or longer before procurement (ie, >7 days) or a positive antibody test result were considered to have a resolved COVID-19 infection. Overall, 35 462 of 35 851 donors (98.9%) with NAT results were tested within 30 days before procurement.

### Outcomes

Kidney nonuse was defined as a kidney recovered for transplant but not transplanted. Primary KT outcomes were all-cause kidney allograft failure and patient death. For kidney allograft failure, the survival time was calculated from the date of transplant to the date of graft failure defined by a return to dialysis, kidney retransplant, or patient death. For patient death, patients were followed up until death or date of censoring. Secondary KT outcomes were acute rejection (ie, rejection in the first 6 months after KT); transplant hospitalization length of stay (LOS) between KT and discharge; and delayed graft function (DGF), defined as receipt of dialysis in the first week after KT.

### Covariates

Donor characteristics included age, sex, race and ethnicity, body mass index (calculated as weight in kilograms divided by height in meters squared), presence of diabetes, presence of hypertension, kidney donor profile index score, donation after cardiac death status, cause of death, serum creatinine level, and hepatitis C virus status. Recipient characteristics included age at transplant, sex, race and ethnicity (including Black, Hispanic, White, and other [non-Hispanic American Indian or Alaska Native, non-Hispanic Asian, non-Hispanic Native Hawaiian or other Pacific Islander, non-Hispanic multiple races, and unknown non-Hispanic race]), educational attainment, insurance status (private, Medicaid, Medicare, or other), body mass index, presence of diabetes, dialysis status (including duration) at transplant, peak panel reactive antibody level, organ sharing type (local, reginal, or national), days on waiting list before KT, and year of transplant. Race and ethnicity data for donors and recipients were collected because racial and ethnic disparities pervade health care delivery and transplant outcomes for adult and pediatric patients. Data on blood type compatibility, human leukocyte antigen matching, and cytomegalovirus concordance status with the donor, along with cold ischemia time, were also analyzed as covariates. To account for the potential role of the center in which the KT was performed, the center volume of KTs (categorized as <10, 10-19, 20-35, or >35) from COVID-19–positive donors was analyzed as a covariate.

### Statistical Analysis

Summary statistics on donor and recipient characteristics stratified by donor COVID-19 status were calculated. Multivariable logistic regression analysis was conducted to examine the association between donor COVID-19 status and kidney use, adjusting for all donor characteristics. The interaction of COVID-19 status and kidney procurement date was assessed to explore the patterns in nonuse rates for donor COVID-19–positive status over time.

For transplant outcomes, we first used Kaplan-Meier analyses to compare graft failure by donor COVID-19 status. Multivariable Cox regression analysis was used to estimate adjusted hazard ratios (AHRs) for all-cause graft failure and patient death associated with donor COVID-19 status, adjusting for donor and recipient characteristics. We used linear regression analysis to compare LOS by donor COVID-19 status and logistic regression analysis to compare the adjusted odds ratios (AORs) of DGF and acute rejection by donor COVID-19 status.

To control for patient selection to receive a kidney from a COVID-19–positive donor, we weighted all models inversely proportional to propensity scores generated by multinomial logistic regression analysis. Balance in recipient characteristics by donor COVID-19 status was evaluated by calculating standardized differences in the weighted sample. Groups were considered balanced when the absolute value was smaller than 0.1 (eTable 1 in Supplement 1).

All tests of statistical significance were 2-sided, and *P* < .05 was considered statistically significant. Analyses were performed using Stata software, version 17 SE (StataCorp LLC).

## Results

### Kidney Use

In total, 71 334 kidneys were recovered from 35 851 deceased donors with COVID-19 test results from March 1, 2020, to March 30, 2023. Among those, the mean (SD) age was 42.5 (15.3) years; 22 319 (62.3%) were men, 13 532 (37.7%) were women, and 23 992 (66.9%) were White. Summary statistics on donor characteristics of recovered kidneys by COVID-19 status are shown in Table 1. The median (IQR) time from COVID-19 diagnosis to KT was 24 (12-43) days in those with resolved infection and 3 (2-5) days in those with active infection. Of 66 831 recovered kidneys from COVID-19–negative donors, 16 175 (24.2%) were not used and 50 656 (75.8%) were transplanted. Of 2165 recovered kidneys from active COVID-19–positive donors, 632 (29.2%) were not used and 1533 (70.8%) were transplanted. Of 2338 recovered kidneys from resolved COVID-19–positive donors, 615 (26.3%) were not used, and 1723 (73.7%) were transplanted. Donor characteristics of unused kidneys are summarized in eTable 2 in Supplement 1.

**Table 1.** Donor Characteristics of Kidneys Recovered for Transplant From March 1, 2020, to March 30, 2023, by Donor COVID-19 Status

Characteristic	Kidneys, No./total No. (%)^a^			*P* value^b^
	From COVID-19–negative donors (n = 66 831)	From donors with active COVID-19 (n = 2165)	From donors with resolved COVID-19 (n = 2338)	
Total donors, No.	33 591	1089	1171	NA
Kidney disposition^c^^,^^d^				
Not used	16 175/66 831 (24.2)	632/2165 (29.2)	615/2338 (26.3)	<.001
Transplanted	50 656/66 831 (75.8)	1533/2165 (70.8)	1723/2338 (73.7)	
Recovery year^c^^,^^d^				
2020 (March to December)	13 240/66 831 (19.8)	10/2165 (0.5)	24/2338 (1.0)	<.001
2021	23 634/66 831 (35.4)	375/2165 (17.3)	541/2338 (23.1)	
2022	23 886/66 831 (35.7)	1488/2165 (68.7)	1462/2338 (62.5)	
2023 (January to March)	6071/66 831 (9.1)	292/2165 (13.5)	311/2338 (13.3)	
Donor characteristics				
Age, y^c^				
<18	3320/66 831 (5.0)	123/2165 (5.7)	120/2338 (5.1)	.12
18-30	12 901/66 831 (19.3)	438/2165 (20.2)	457/2338 (19.5)	
31-44	19 101/66 831 (28.6)	612/2165 (28.3)	680/2338 (29.1)	
45-59	21 605/66 831 (32.3)	705/2165 (32.6)	778/2338 (33.3)	
≥60	9904/66 831 (14.8)	287/2165 (13.3)	303/2338 (13.0)	
Sex				
Female	25 241/66 831 (37.8)	817/2165 (37.7)	878/2338 (37.6)	.98
Male	41 590/66 831 (62.2)	1348/2165 (62.3)	1460/2338 (62.4)	
Race and ethnicity^c^^,^^d^				
Black	9863/66 831 (14.8)	286/2165 (13.2)	338/2338 (14.5)	<.001
Hispanic	9551/66 831 (14.3)	303/2165 (14.0)	465/2338 (19.9)	
White	44 810/66 831 (67.0)	1497/2165 (69.1)	1425/2338 (60.9)	
Other^e^	2607/66 831 (3.9)	79/2165 (3.6)	110/2338 (4.7)	
BMI^c^				
<18.5, underweight	2230/66 612 (3.3)	76/2160 (3.5)	104/2325 (4.5)	<.001
18.5-24.9, normal weight	19 822/66 612 (29.8)	591/2160 (27.4)	618/2325 (26.6)	
25.0-29.9, overweight	19 805/66 612 (29.7)	593/2160 (27.5)	651/2325 (28.0)	
30.0-34.9, obesity class 1	12 884/66 612 (19.3)	474/2160 (21.9)	458/2325 (19.7)	
35.0-39.9, obesity class 2	6449/66 612 (9.7)	210/2160 (9.7)	274/2325 (11.8)	
≥40.0, obesity class 3	5422/66 612 (8.1)	216/2160 (10.0)	220/2325 (9.5)	
Diabetes^d^				
No	57 440/65 784 (87.3)	1878/2128 (88.3)	2003/2306 (86.9)	.35
Yes	8344/65 784 (12.7)	250/2128 (11.7)	303/2306 (13.1)	
Hypertension^c^				
No	41 933/65 739 (63.8)	1428/2124 (67.2)	1543/2304 (67.0)	<.001
Yes	23 806/65 739 (36.2)	696/2124 (32.8)	761/2304 (33.0)	
Kidney donor profile index, %^c^				
<25	16 367/66 831 (24.5)	552/2165 (25.5)	602/2338 (25.7)	<.001
25-49	17 097/66 831 (25.6)	586/2165 (27.1)	662/2338 (28.3)	
50-84	22 714/66 831 (34.0)	745/2165 (34.4)	779/2338 (33.3)	
≥85	10 653/66 831 (15.9)	282/2165 (13.0)	295/2338 (12.6)	
Donation after cardiac death^c^^,^^d^				
No	44 789/64 728 (69.2)	1407/2077 (67.7)	1413/2256 (62.6)	<.001
Yes	19 939/64 728 (30.8)	670/2077 (32.3)	843/2256 (37.4)	
Cause of death^c^^,^^d^				
Anoxia	31 856/66 831 (47.7)	979/2165 (45.2)	1095/2338 (46.8)	<.001
Cerebrovascular disease or stroke	16 198/66 831 (24.2)	490/2165 (22.6)	506/2338 (21.6)	
Head trauma	16 356/66 831 (24.5)	465/2165 (21.5)	445/2338 (19.0)	
CNS tumor	216/66 831 (0.3)	2/2165 (0.1)	12/2338 (0.5)	
Other	2205/66 831 (3.3)	229/2165 (10.6)	280/2338 (12.0)	
Serum creatinine level, mg/dL^c^^,^^d^				
<1.0	31 790/64 207 (49.5)	1060/2049 (51.7)	1316/2236 (58.9)	<.001
1.0-1.5	14 232/64 207 (22.2)	416/2049 (20.3)	426/2236 (19.1)	
>1.5	18 185/64 207 (28.3)	573/2049 (28.0)	494/2236 (22.1)	
Donor HCV status^c^				
Ab negative and NAT negative	59 521/66 789 (89.1)	1925/2161 (89.1)	2126/2338 (90.9)	.02
Ab positive and NAT negative	3183/66 789 (4.8)	120/2161 (5.6)	92/2338 (3.9)	
NAT positive	4085/66 789 (6.1)	116/2161 (5.4)	120/2338 (5.1)	

Abbreviations: Ab, antibody; BMI, body mass index (calculated as weight in kilograms divided by height in meters squared); CNS, central nervous system; HCV, hepatitis C virus; NA, not applicable; NAT, nucleic acid amplification test.

SI conversion factor: To convert serum creatine from milligrams per deciliter to micromoles per liter, multiply by 88.4.

^a^ Denominators vary due to missing values.

^b^ *P* values for testing differences between COVID-19–negative, active COVID-19–positive, and resolved COVID**-**19–positive groups.

^c^ Significance threshold was *P* < .05 for χ^2^ tests comparing differences between COVID-19–positive and COVID-19–negative groups.

^d^ Significance threshold was *P* < .05 for χ^2^ tests comparing differences between active COVID-19–positive and resolved COVID-19–positive groups.

^e^ Includes non-Hispanic American Indian or Alaska Native, non-Hispanic Asian, non-Hispanic Native Hawaiian or other Pacific Islander, non-Hispanic multiple races, and unknown non-Hispanic race.

Compared with kidneys from COVID-19–negative donors, kidneys from active COVID-19–positive donors had 56% higher odds of nonuse (AOR, 1.55; 95% CI, 1.38-1.76), while kidneys from donors with resolved COVID-19 had 31% higher odds of nonuse (AOR, 1.31; 95% CI, 1.16-1.48). Adding the interaction term of donor COVID-19 status and kidney procurement year significantly improved the fit of the model (likelihood ratio χ^2^ = 32.51; *P* < .001). The likelihood of nonuse of kidneys from active or resolved COVID-19–positive donors decreased over time (Table 2). Kidneys from active COVID-19–positive donors had approximately 11-fold higher odds of nonuse in 2020 (AOR, 11.26; 95% CI, 2.29-55.38), 2-fold higher odds of nonuse in 2021 (AOR, 2.09; 95% CI, 1.58-2.79), and 1.5-fold higher odds of nonuse in 2022 (AOR, 1.47; 95% CI, 1.28-1.70). However, kidneys from active COVID-19–positive donors procured in 2023 were not associated with higher odds of nonuse (AOR, 1.07; 95% CI, 0.75-1.63). Kidneys from resolved COVID-19 –positive donors had approximately 4-fold higher odds of nonuse in 2020 (AOR, 3.87; 95% CI, 1.26-11.90), 2-fold higher odds of nonuse in 2021 (AOR, 1.94; 95% CI, 1.54-2.45), and no higher odds of nonuse in 2022 (AOR, 1.09; 95% CI, 0.94-1.28) and 2023 (AOR, 1.18; 95% CI, 0.80-1.73).

**Table 2.** Nonuse of Kidneys Recovered From Active and Resolved COVID-19–Positive Donors, Organ Procurement and Transplantation Network 2020-2023

COVID-19 status	AOR (95% CI)^a^				
	Overall	2020 (March to December)	2021	2022	2023 (January to March)
COVID-19 negative	1 [Reference]	1 [Reference]	1 [Reference]	1 [Reference]	1 [Reference]
Active COVID-19 positive	1.55 (1.38-1.76)	11.26 (2.29-55.38)	2.09 (1.58-2.79)	1.47 (1.28-1.70)	1.07 (0.75-1.63)
Resolved COVID-19 positive	1.31 (1.16-1.48)	3.87 (1.26-11.90)	1.94 (1.54-2.45)	1.09 (0.94-1.28)	1.18 (0.80-1.73)

Abbreviation: AOR, adjusted odds ratio.

^a^ Adjusted for donor characteristics, including age, sex, race and ethnicity, body mass index, presence of diabetes, presence of hypertension, kidney donor profile index score, donation after cardiac death status, cause of death, serum creatinine level, and hepatitis C virus status.

### Transplant Outcomes

A total of 45 912 adult KT recipients (mean [SD] age, 54.3 [13.2] years; 27 952 [60.9%] male, 17 960 [39.1%] female, and 15 349 [33.4%] Black) were included in the outcome analysis. Summary statistics on recipient characteristics by COVID-19 status are shown in eTable 3 in Supplement 1. In unadjusted analyses, no association was observed between donor COVID-19 status and graft failure over 2 years of follow-up (Figure). The median (IQR) follow-up was 200 (15-355) days for the active or resolved COVID-19–positive group. The multivariable Cox regression analysis revealed that recipients of kidneys from donors with active COVID-19 did not have a higher risk of graft failure (AHR, 1.03; 95% CI, 0.78-1.37) or patient death (AHR, 1.17; 95% CI, 0.84-1.66) compared with recipients of kidneys from COVID-19–negative donors (Table 3). Similarly, recipients of kidneys from donors with resolved COVID-19 did not have a higher risk of graft failure (AHR, 1.10; 95% CI, 0.88-1.39) or patient death (AHR, 0.95; 95% CI, 0.70-1.28) compared with recipients of kidneys from COVID-19–negative donors (Table 3).

**Figure.**
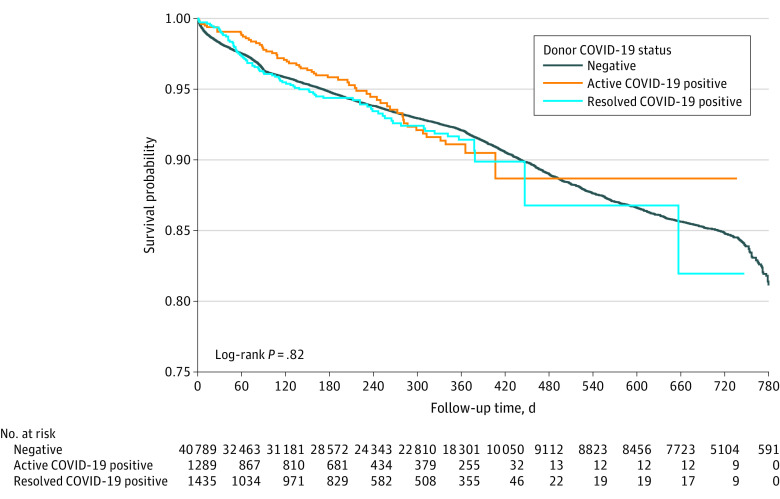
Kaplan-Meier Curve for Graft Failure by Donor COVID-19 Status

**Table 3.** Transplant Outcomes of Patients Receiving Kidneys From Active and Resolved COVID-19–Positive Deceased Donors^a^

COVID-19 status	AHR (95% CI)^b^	
	All-cause kidney graft failure	All-cause patient death
COVID-19 negative	1 [Reference]	1 [Reference]
Active COVID-19 positive	1.03 (0.78-1.37)	1.17 (0.84-1.66)
Resolved COVID-19 positive	1.10 (0.88-1.39)	0.95 (0.70-1.28)

Abbreviation: AHR, adjusted hazard ratio.

^a^ Results of multivariable Cox regression model with inverse probability of treatment weighting.

^b^ Adjusted for donor characteristics (age, sex, race and ethnicity, body mass index, presence of diabetes, presence of hypertension, donation after cardiac death, and kidney donor profile index score), recipient characteristics (age, sex, race and ethnicity, educational attainment, insurance status, body mass index, presence of diabetes, dialysis duration, peak panel reactive antibody level, organ share type, and days on waiting list before transplant), and transplant characteristics (blood type compatibility, human leukocyte antigen matching, cytomegalovirus concordance status with donors, cold ischemia time, center volume of kidney transplants from COVID-19 positive donors, and year of transplant).

No association with higher risk of acute rejection within 6 months after KT was observed among recipients of kidneys from donors with active COVID-19 (AOR, 0.99; 95% CI, 0.66-1.48) or resolved COVID-19 (AOR, 0.72; 95% CI, 0.47-1.09). Neither kidneys from active COVID-19–positive donors (AOR, 0.92; 95% CI, 0.79-1.05) nor resolved COVID-19–positive donors (AOR, 1.03; 95% CI, 0.91-1.17) were associated with a higher risk of DGF. Recipients of kidneys from active COVID-19–positive donors had 0.63 days (95% CI, 0.23-1.01 days; *P* = .002) shorter LOS than recipients of kidneys from COVID-19–negative donors.

## Discussion

In this cohort study of 35 831 deceased donors and 45 912 adult KT recipients, we found a pattern of lower likelihood of kidney nonuse from deceased donors with active or resolved COVID-19 over time. In 2020, kidneys procured from active COVID-19–positive donors had an 11-fold higher likelihood of nonuse, while those from resolved COVID-19–positive donors had a 4-fold higher probability of nonuse compared with kidneys from COVID-19–negative donors. In 2023, kidneys from either active or resolved COVID-19–positive deceased donors were no longer associated with higher risk of nonuse. In addition, among adult KT recipients, we found that kidneys from donors with resolved or active COVID-19 were not associated with increased risk of graft loss, death, acute rejection, DGF, or longer hospitalization compared with kidneys from COVID-19–negative donors. Our findings provide evidence that the use of kidneys from COVID-19–positive donors yields safe medium-term outcomes, which may be useful information for KT professionals and patients.

The start of the COVID-19 pandemic led to a sharp downturn in the number of organs transplanted in the US, with a 15% reduction in global transplant rates, which favored the procurement of only kidneys with lower kidney donor profile index scores.^10^ This reduction in transplant rates was partially due to redistribution and redeployment of essential personnel, lack of hospital bed availability, and diversion of resources to frontline services, but it was also related to uncertainty about the risk of proceeding with KT in unprecedented times.^16^ However, findings of a previous study^16^ suggest that the benefit of proceeding with KT during a global pandemic can be preserved when hospitals have adequate resources and that there may be substantial lost opportunities from not using available organs for transplant during a pandemic. While the risk-benefit profile of using kidneys obtained from COVID-19–positive donors is less established, the current study found that there was a higher rate of nonuse of kidneys from COVID-19–positive donors.

Given that more than 40% of individuals in the US had evidence of a past COVID-19 diagnosis as of May 2022,^17^ excluding potential kidney donors based on past or current COVID-19 diagnosis would substantially limit opportunities for organ use and KT, which is not a benign consequence. From 2008 to 2015, patients on the US KT waiting list experienced a higher likelihood of dying or withdrawing from the waiting list (30.5%) than undergoing transplant (29.2%), with deaths per day of approximately 10 candidates who had at least 1 previous allograft offer declined.^18^ Therefore, maximizing the use of all potentially transplantable organs is a life-saving priority.

Early evidence from the beginning of the pandemic^19,20,21^ suggests that proceeding with transplant of solid organs retrieved from living and deceased donors after confirmation of resolution of COVID-19 is likely safe. However, other studies^12,22,23,24^ have recommended against transplant of organs from donors with active COVID-19 given direct evidence of transmission in the case of lung transplant and laboratory evidence of plausible transmission from other organs. For KT, successful transplant using kidneys from donors with active COVID-19 has been reported,^20,25,26,27^ and data have revealed no increase in adverse outcomes and no donor-derived infections with these kidney allografts.^20,21,25^ A recent study^28^ of 423 COVID-19–positive donors in the US from March 12, 2020, to August 31, 2021, found 2-fold higher adjusted odds of nonuse of kidneys from SARS-CoV-2–positive donors and comparable 6-month graft survival among 353 kidneys from COVID-19–positive donors compared with kidneys from COVID-19–negative donors. While that study^28^ was an important first step in examining medium-term KT outcomes among those receiving kidneys from COVID-19–positive deceased donors, the adjusted change in nonuse rate over time was not examined, and there was no analysis of donors with resolved COVID-19. To our knowledge, our analysis of national registry data up to March 30, 2023, included the largest sample of COVID-19–positive donors and recipients to date, and our analysis of 2-year KT outcomes represented the longest follow-up period reported to date.

We found that the likelihood of nonuse of kidneys from active COVID-19–positive donors decreased over time. For kidneys procured in 2023, donor COVID-19 positivity was not associated with higher risk of nonuse. As the COVID-19 pandemic progressed, various factors may have played a role in these observed patterns, including enhanced institutional operations and resource allocation, the introduction of novel treatment options, the development and distribution of vaccines, and the establishment of guidance or published recommendations regarding clinical practice and management within the solid organ transplant community. Moreover, improvements in both the accessibility and performance of COVID-19 diagnostic testing facilitated routine screening and evaluation of potential organ donors.

National guidelines from the American Society of Transplantation were last updated in January 2023^29^ and state that donors who are SARS-CoV-2 NAT-positive and who died of COVD-19–attributable complications should be considered for nonlung transplant acceptance, acknowledging limited long-term outcome data availability. Our findings support the use of these valuable organs and may encourage organ procurement organizations to consider recovering more kidneys from COVID-19–positive donors and promote further acceptance among transplant professionals. Although the number of COVID-19 infections is decreasing, testing for the virus in donors remains mandatory. This study could potentially help the transplant community consider discontinuing COVID-19 testing for donors in the near future. Our findings offer robust evidence suggesting that the use of kidneys from COVID-19–positive donors is safe during medium-term follow-up; however, longer-term follow-up is necessary to further validate this practice.

### Limitations

This study has several limitations. Due to the lack of a timeline for SARS-CoV-2 infection among donors, we determined active or resolved status based on test specimen collection date and donor recovery date. Second, this study was retrospective and thus can identify associations but not establish causation; therefore, the results should be interpreted carefully. Third, selection bias may have occurred based on whether candidates chose to receive kidneys from COVID-19–positive donors. While we tried to account for this potential bias in our analysis by using propensity score weightings to improve covariate balance across the groups, it is possible that residual confounding remained based on decision-making regarding which COVID-19–positive donors to accept. Fourth, due to the relative recency of the pandemic and the fact that the first kidneys from COVID-19–positive donors were transplanted in August 2020, we had only 2 years (median, 200 days) of follow-up for COVID-19–positive KTs. Therefore, our analyses cannot exclude the possibility that kidneys from COVID-19–positive donors may confer an increased risk of graft loss or death beyond 2 years.

## Conclusions

This cohort study found that the likelihood of nonuse of COVID-19–positive donor kidneys decreased over time and, for kidneys procured in 2023, donor COVID-19 positivity was no longer associated with higher odds of nonuse. Transplant of kidneys from donors with resolved or active COVID-19 was not associated with increased risk of all-cause graft loss, all-cause death, acute rejection, DGF, or longer hospitalization over more than 2 years of follow-up compared with kidneys from COVID-19–negative donors. These findings suggest that the use of kidneys from donors with COVID-19 is safe in the medium term. Further research is needed to assess longer-term transplant outcomes involving kidneys from COVID-19–positive donors.

## Notes

Supplement 1. **eTable 1.** Absolute Standardized Differences of Kidney Transplant Recipient Characteristics Between Weighted Samples **eTable 2.** Donor Characteristics for Kidneys Recovered for Transplant From March 1, 2020, to March 30, 2023, by Donor COVID-19 Status and Kidney Disposition **eTable 3.** Characteristics of Recipients by Deceased Kidney Donor COVID-19 Status, OPTN 2020-2023

Supplement 2.
Data Sharing Statement

## References

[1] Aubert O, Yoo D, Zielinski D, . COVID-19 pandemic and worldwide organ transplantation: a population-based study. Lancet Public Health. 2021;6(10):e709-e719. doi:10.1016/S2468-2667(21)00200-0 34474014 PMC8460176

[2] Goff RR, Wilk AR, Toll AE, McBride MA, Klassen DK. Navigating the COVID-19 pandemic: initial impacts and responses of the Organ Procurement and Transplantation Network in the United States. Am J Transplant. 2021;21(6):2100-2112. doi:10.1111/ajt.16411 33244847 PMC7754561

[3] Loupy A, Aubert O, Reese PP, Bastien O, Bayer F, Jacquelinet C. Organ procurement and transplantation during the COVID-19 pandemic. Lancet. 2020;395(10237):e95-e96. doi:10.1016/S0140-6736(20)31040-0 32407668 PMC7213957

[4] Lentine KL, Mannon RB, Josephson MA. Practicing with uncertainty: kidney transplantation during the COVID-19 pandemic. Am J Kidney Dis. 2021;77(5):777-785. doi:10.1053/j.ajkd.2020.12.003 33388404 PMC7946342

[5] Wang JH, Hart A. Global perspective on kidney transplantation: United States. Kidney360. 2021;2(11):1836-1839. doi:10.34067/KID.0002472021 35373000 PMC8785833

[6] Lentine KL, Smith JM, Hart A, . OPTN/SRTR 2020 annual data report: kidney. Am J Transplant. 2022;22(suppl 2):21-136. doi:10.1111/ajt.16982 35266618

[7] Boyarsky BJ, Po-Yu Chiang T, Werbel WA, . Early impact of COVID-19 on transplant center practices and policies in the United States. Am J Transplant. 2020;20(7):1809-1818. doi:10.1111/ajt.15915 32282982 PMC7262146

[8] Ahn C, Amer H, Anglicheau D, . Global transplantation COVID report March 2020. Transplantation. 2020;104(10):1974-1983. doi:10.1097/TP.0000000000003258 32243281 PMC7188045

[9] Gupta G, Azhar A, Gungor A, Molnar MZ, Morales MK, Tanriover B. Early data on utilization and discard of organs from COVID-19–infected donors: a US National Registry analysis. Transplantation. 2022;106(5):e266-e268. doi:10.1097/TP.0000000000004091 35250008 PMC9038239

[10] Li MT, King KL, Husain SA, Schold JD, Mohan S. Deceased donor kidneys utilization and discard rates during COVID-19 pandemic in the United States. Kidney Int Rep. 2021;6(9):2463-2467. doi:10.1016/j.ekir.2021.06.002 34514207 PMC8419126

[11] Dhand A, Gass A, John D, . Long-term and short-term outcomes of solid organ transplantation from donors with a positive SARS-CoV-2 test. Transplantation. 2022;106(8):e384-e385. doi:10.1097/TP.0000000000004196 35575769 PMC9311281

[12] Stein SR, Ramelli SC, Grazioli A, ; NIH COVID-19 Autopsy Consortium. SARS-CoV-2 infection and persistence in the human body and brain at autopsy. Nature. 2022;612(7941):758-763. doi:10.1038/s41586-022-05542-y 36517603 PMC9749650

[13] Organ Procurement & Transplantation Network. National data: donors recovered in the U.S. by donor type. Health Resources & Services Administration, US Dept of Health & Human Services. Accessed February 10, 2023. https://optn.transplant.hrsa.gov/data/view-data-reports/national-data/#

[14] Massie AB, Kucirka LM, Segev DL. Big data in organ transplantation: registries and administrative claims. Am J Transplant. 2014;14(8):1723-1730. doi:10.1111/ajt.12777 25040084 PMC4387865

[15] Organ Procurement & Transplantation Network. About data. Health Resources & Services Administration, US Dept of Health & Human Services. Accessed April 10, 2023. https://optn.transplant.hrsa.gov/data/about-data/

[16] Vinson AJ, Kiberd BA, Tennankore KK. Panic in the pandemic: when should kidney transplant programs close? Kidney Int Rep. 2021;6(5):1232-1241. doi:10.1016/j.ekir.2021.02.017 34013101 PMC8116904

[17] Akinbami LJ. Kruszon-Moran D, Wang CY, et al. SARS-CoV-2 serology and self-reported infection among adults—National Health and Nutrition Examination Survey, United States, August 2021–May 2022. *MMWR Morb Mortal Wkly Rep*. 2022;71(48):1522-1525. doi:10.15585/mmwr.mm7148a4 PMC9721142 36454698

[18] Husain SA, King KL, Pastan S, . Association between declined offers of deceased donor kidney allograft and outcomes in kidney transplant candidates. JAMA Netw Open. 2019;2(8):e1910312. doi:10.1001/jamanetworkopen.2019.10312 31469394 PMC6724162

[19] Weiss MJ, Hornby L, Foroutan F, . Clinical practice guideline for solid organ donation and transplantation during the COVID-19 pandemic. Transplant Direct. 2021;7(10):e755. doi:10.1097/TXD.0000000000001199 34514110 PMC8425831

[20] Kute VB, Fleetwood VA, Meshram HS, Guenette A, Lentine KL. Use of organs from SARS-CoV-2 infected donors: is it safe? a contemporary review. Curr Transplant Rep. 2021;8(4):281-292. doi:10.1007/s40472-021-00343-0 34722116 PMC8546195

[21] Neidlinger NA, Smith JA, D’Alessandro AM, . Organ recovery from deceased donors with prior COVID-19: a case series. Transpl Infect Dis. 2021;23(2):e13503. doi:10.1111/tid.13503 33174324 PMC8244092

[22] Kaul DR, Valesano AL, Petrie JG, . Donor to recipient transmission of SARS-CoV-2 by lung transplantation despite negative donor upper respiratory tract testing. Am J Transplant. 2021;21(8):2885-2889. doi:10.1111/ajt.16532 33565705 PMC8014875

[23] Kumar D, Humar A, Keshavjee S, Cypel M. A call to routinely test lower respiratory tract samples for SARS-CoV-2 in lung donors. Am J Transplant. 2021;21(7):2623-2624. doi:10.1111/ajt.16576 33756058 PMC8251114

[24] Gaussen A, Hornby L, Rockl G, . Evidence of SARS-CoV-2 infection in cells, tissues, and organs and the risk of transmission through transplantation. Transplantation. 2021;105(7):1405-1422. doi:10.1097/TP.0000000000003744 33724248

[25] Safa K, Elias N, Gilligan HM, Kawai T, Kotton CN. Successful living kidney donation after COVID-19 infection. Transplantation. 2021;105(1):e4-e5. doi:10.1097/TP.0000000000003510 33350629

[26] Puodziukaite L, Serpytis M, Kundrotaite A, . Kidney transplantation from a SARS-CoV-2–positive donor for the recipients with immunity after COVID-19. Transpl Infect Dis. 2021;23(4):e13666. doi:10.1111/tid.13666 34097791 PMC8209852

[27] Frattaroli P, Anjan S, Coro A, . Is it safe to perform abdominal transplantation from SARS-CoV-2 polymerase chain reaction positive donors? Transpl Infect Dis. 2021;23(5):e13688. doi:10.1111/tid.13688 34258844 PMC8420592

[28] Schold JD, Koval CE, Wee A, Eltemamy M, Poggio ED. Utilization and outcomes of deceased donor SARS-CoV-2–positive organs for solid organ transplantation in the United States. Am J Transplant. 2022;22(9):2217-2227. doi:10.1111/ajt.17126 35730252 PMC9350307

[29] American Society of Transplantation. SARS-CoV-2: recommendations and guidance for organ donor testing and evaluation. American Society of Transplantation. Updated January 18, 2023. Accessed April 10, 2023. https://www.myast.org/sites/default/files/Donor%20Testing%20Document1.18.23.pdf

