# Supplementary material for: Patterns in Use and Transplant Outcomes Among Adult Recipients of Kidneys From Deceased Donors With COVID-19
Source: JAMA Netw Open. 2023 May 30;6(5):e2315908. doi: 10.1001/jamanetworkopen.2023.15908 (PMC10230314; doi:10.1001/jamanetworkopen.2023.15908)
Supplement: Supplement 1. — eTable 1. Absolute Standardized Differences of Kidney Transplant Recipient Characteristics Between Weighted Samples eTable 2. Donor Characteristics for Kidneys Recovered for Transplant From March 1, 2020, to March 30, 2023, by Donor COVID-19 Status and Kidney Disposition eTable 3. Characteristics of Recipients by Deceased Kidney Donor COVID-19 Status, OPTN 2020-2023 [file jamanetwopen-e2315908-s001.pdf]

Supplementary Online Content

Ji M, Vinson AJ, Chang SH, et al. Patterns in use and transplant outcomes among adult recipients of kidneys from deceased donors with COVID-19. *JAMA Netw Open*. 2023;6(5):e2315908. doi:10.1001/jamanetworkopen.2023.15908

eTable 1. Absolute Standardized Differences of Kidney Transplant Recipient Characteristics Between Weighted Samples

eTable 2. Donor Characteristics for Kidneys Recovered for Transplant From March 1, 2020, to March 30, 2023, by Donor COVID-19 Status and Kidney Disposition

eTable 3. Characteristics of Recipients by Deceased Kidney Donor COVID-19 Status, OPTN 2020-2023

This supplemental material has been provided by the authors to give readers additional information about their work.

© 2023 Ji M et al. *JAMA Network Open*.

eTable 1. Absolute Standardized Differences of Kidney Transplant Recipient Characteristics Between Weighted Samples

Recipient characteristics	COVID-19 negative VS Active COVID-19+	COVID-19 negative VS Resolved COVID-19+	Active COVID-19+ VS Resolved COVID-19+
Age	0.003	0.009	0.005
Male sex (%)	0.020	0.009	0.028
Race/ethnicity (%)			
African-American	0.001	0.009	0.009
Caucasian	0.041	0.007	0.034
Hispanic	0.017	0.006	0.023
Other	0.039	0.005	0.034
Education (%)			
Less than high school	0.033	0.015	0.018
High school	0.006	0.006	0.000
College	0.009	0.006	0.003
Bachelor's degree	0.007	0.004	0.004
Graduate degree	0.003	0.009	0.012
Insurance (%)			
Private	0.004	0.004	0.000
Medicaid/CHIP	0.021	0.009	0.030
Medicare/government	0.027	0.002	0.029
Other	0.031	0.002	0.033
Body mass index (%)			
<18.5	0.014	0.019	0.033
18.5-24.9	0.005	0.004	0.001
25-29.9	0.008	0.007	0.000
≥30	0.019	0.011	0.030
History of diabetes (%)	0.012	0.008	0.022
Dialysis duration	0.030	0.000	0.012
0% peak panel reactive antibody level (%)	0.026	0.002	0.028
Organ share type (%)			
Local	0.005	0.001	0.006
Reginal	0.015	0.003	0.018
National	0.009	0.002	0.011
Time in Waitlist	0.015	0.018	0.014

© 2023 Ji M et al. *JAMA Network Open*.

eTable 2. Donor Characteristics for Kidneys Recovered for Transplant From March 1, 2020, to March 30, 2023, by Donor COVID-19 Status and Kidney Disposition

	Utilized kidneys			Non-utilized kidneys		
	COVID-19 negative (n=50656)	Active COVID-19 (n=1533)	Resolved COVID-19 (n=1723)	COVID-19 negative (n=16,175)	Active COVID-19 (n=632)	Resolved COVID-19 (n=615)
Age (year)						
<18	3138 (6.19%)	112 (7.31%)	114 (6.62%)	182 (1.13%)	11 (1.74%)	6 (0.98%)
18-30	12063 (23.81%)	409 (26.68%)	418 (24.26%)	838 (5.18%)	29 (4.59%)	39 (6.34%)
31-44	16759 (33.08%)	512 (33.4%)	589 (34.18%)	2342 (14.48%)	100 (15.82%)	91 (14.8%)
45-59	14767 (29.15%)	417 (27.2%)	506 (29.37%)	6838 (42.28%)	288 (45.57%)	272 (44.23%)
>=60	3929 (7.76%)	83 (5.41%)	96 (5.57%)	5975 (36.94%)	204 (32.28%)	207 (33.66%)
Sex						
Female	18214 (35.96%)	532 (34.7%)	609 (35.35%)	7027 (43.44%)	285 (45.09%)	269 (43.74%)
Male	32442 (64.04%)	1001 (65.3%)	1114 (64.65%)	9148 (56.56%)	347 (54.91%)	346 (56.26%)
Race/ethnicity						
White	33768 (66.66%)	1051 (68.56%)	1030 (59.78%)	11042 (68.27%)	446 (70.57%)	395 (64.23%)
Black	7258 (14.33%)	187 (12.2%)	252 (14.63%)	2605 (16.11%)	99 (15.66%)	86 (13.98%)
Hispanic	7658 (15.12%)	236 (15.39%)	361 (20.95%)	1893 (11.7%)	67 (10.6%)	104 (16.91%)
Other	1972 (3.89%)	59 (3.85%)	80 (4.64%)	635 (3.93%)	20 (3.16%)	30 (4.88%)
Body Mass Index (kg/m^2)						
Underweight	1762 (3.49%)	62 (4.05%)	76 (4.44%)	468 (2.91%)	14 (2.23%)	28 (4.58%)
Normal	15900 (31.47%)	486 (31.72%)	500 (29.19%)	3922 (24.39%)	105 (16.72%)	118 (19.28%)
Overweight	15275 (30.23%)	404 (26.37%)	471 (27.5%)	4530 (28.17%)	189 (30.1%)	180 (29.41%)
Obese I	9460 (18.72%)	310 (20.23%)	324 (18.91%)	3424 (21.29%)	164 (26.11%)	134 (21.9%)
Obese II	4573 (9.05%)	139 (9.07%)	197 (11.5%)	1876 (11.66%)	71 (11.31%)	77 (12.58%)
Obese III	3559 (7.04%)	131 (8.55%)	145 (8.46%)	1863 (11.58%)	85 (13.54%)	75 (12.25%)
Diabetes						
No	45977 (92.12%)	1397 (92.95%)	1552 (90.97%)	11463 (72.2%)	481 (76.96%)	451 (75.17%)
Yes	3931 (7.88%)	106 (7.05%)	154 (9.03%)	4413 (27.8%)	144 (23.04%)	149 (24.83%)
Hypertension						
No	36182 (72.56%)	1160 (77.13%)	1283 (75.16%)	5751 (36.23%)	268 (43.23%)	260 (43.55%)
Yes	13683 (27.44%)	344 (22.87%)	424 (24.84%)	10123 (63.77%)	352 (56.77%)	337 (56.45%)

© 2023 Ji M et al. *JAMA Network Open*.

Kidney donor profile index, %						
<25	15795 (31.18%)	535 (34.9%)	569 (33.02%)	572 (3.54%)	17 (2.69%)	33 (5.37%)
25-49	15438 (30.48%)	489 (31.9%)	576 (33.43%)	1659 (10.26%)	97 (15.35%)	86 (13.98%)
50-84	16112 (31.81%)	446 (29.09%)	488 (28.32%)	6602 (40.82%)	299 (47.31%)	291 (47.32%)
>=85	3311 (6.54%)	63 (4.11%)	90 (5.22%)	7342 (45.39%)	219 (34.65%)	205 (33.33%)
Donation after cardiac death						
No	35118 (71.51%)	1048 (71.83%)	1107 (66.69%)	9671 (61.92%)	359 (58.09%)	306 (51.34%)
Yes	13991 (28.49%)	411 (28.17%)	553 (33.31%)	5948 (38.08%)	259 (41.91%)	290 (48.66%)
Cause of death						
Anoxia	24645 (48.65%)	741 (48.34%)	837 (48.58%)	7211 (44.58%)	238 (37.66%)	258 (41.95%)
Cerebrovascular/stroke	10122 (19.98%)	259 (16.89%)	318 (18.46%)	6076 (37.56%)	231 (36.55%)	188 (30.57%)
Head trauma	14183 (28%)	387 (25.24%)	391 (22.69%)	2173 (13.43%)	78 (12.34%)	54 (8.78%)
CNS tumor	161 (0.32%)	2 (0.13%)	8 (0.46%)	55 (0.34%)	0 (0%)	4 (0.65%)
Other	1545 (3.05%)	144 (9.39%)	169 (9.81%)	660 (4.08%)	85 (13.45%)	111 (18.05%)
Serum creatinine						
<1	26816 (55.06%)	830 (57.4%)	1060 (64.28%)	4974 (32.08%)	230 (38.14%)	256 (43.61%)
1-1.5	10989 (22.56%)	314 (21.72%)	310 (18.8%)	3243 (20.92%)	102 (16.92%)	116 (19.76%)
>1.5	10898 (22.38%)	302 (20.89%)	279 (16.92%)	7287 (47%)	271 (44.94%)	215 (36.63%)
HCV donor status						
Ab-/NAT-	45333 (89.54%)	1368 (89.35%)	1564 (90.77%)	14188 (87.81%)	557 (88.41%)	562 (91.38%)
Ab+/NAT-	2249 (4.44%)	82 (5.36%)	65 (3.77%)	934 (5.78%)	38 (6.03%)	27 (4.39%)
NAT+	3049 (6.02%)	81 (5.29%)	94 (5.46%)	1036 (6.41%)	35 (5.56%)	26 (4.23%)

© 2023 Ji M et al. *JAMA Network Open*.

eTable 3. Characteristics of Recipients by Deceased Kidney Donor COVID-19 Status, OPTN 2020-2023

	COVID-19 negative	Active COVID-19	Resolved COVID-19	P-value
**N**	42,997	1,376	1,539	-----
**Recipient characteristics**				
Age (years, mean, SD)	54.23 (13.20)	53.65 (13.30)	53.63 (13.37)	**0.025**
Male sex (%)	60.81	62.57	61.47	0.372
Race/ethnicity (%)				
African-American	35.56	38.15	35.02	0.263
Caucasian	33.52	32.41	31.97	
Hispanic	20.45	19.84	21.64	
Other	10.47	9.59	11.37	
Education (%)				
Less than high school	7.35	5.89	7.15	0.332
High school	38.57	36.48	39.57	
College	25.05	25.58	24.11	
Bachelor's degree	18.72	21.08	18.58	
Graduate degree	7.3	7.78	7.6	
Insurance (%)				
Private	24.39	27.03	25.99	**<0.001**
Medicaid/CHIP	7.37	7.34	8.77	
Medicare/government	63.88	60.03	59.52	
Other	4.36	5.6	5.72	
Body mass index (%)				
<18.5	26.06	24.65	26.28	0.654
18.5-24.9	1.46	1.38	1.3	
25-29.9	33.14	34.4	34.74	
≥30	39.34	39.56	37.67	
History of diabetes (%)	40.41	39.83	39.73	0.793
Dialysis duration (yrs, mean, SD):	4.72 (3.18)	4.13 (2.76)	4.67 (2.95)	**<0.001**
0% peak panel reactive antibody level (%)	59.18	60.43	59.19	0.222
Organ share type (%)				
Local	50.11	39.24	44.25	**<0.001**

© 2023 Ji M et al. *JAMA Network Open*.

Reginal	24.14	24.85	24.95	
National	25.76	35.9	30.8	
Wait list time (days, median, SD)	762.64 (856.83)	673.99 (755.83)	782.446 (879.20)	**<0.001**
**Transplant characteristics:**				
HLA mismatch				
0-1	4.84	3.56	3.72	**0.048**
2-3	17.69	18.6	18.59	
4-6	77.47	77.83	77.69	
Compatible (vs identical) blood type (%)	3.36	1.89	3.77	**0.002**
Donor/recipient CMV serostatus (%):				
D-/R+	12.28	14.1	12.15	**<0.001**
R+	64.23	61.34	62.25	
D+/R-	17.83	18.1	18.39	
Missing	5.66	6.47	7.21	
Cold ischemia time, hours (%)				
<12	17.11	12.06	15.01	**<0.001**
12-24	54.98	54.65	54.84	
>24	23.13	27.54	24.3	
Missing	4.78	5.74	5.85	
Transplant year (%)				
2020	20.81	0.22	0.65	**<0.001**
2021	35.08	17.59	23.13	
2022	35.08	68.82	63.29	
2023	9.02	13.37	12.93	
Center volume of COVID-19+ KT (%)				
<10	25.02	11.19	14.62	**<0.001**
10-19	24.02	20.49	21.38	
20-35	26.43	28.71	26.19	
>35	24.53	39.61	37.82	
**Donor characteristics:**				
Age (yrs, mean, SD)	39.95 (14.67)	38.01 (14.52)	38.89 (13.93)	**<0.001**
Male sex (%)	63.59	64.68	64.39	0.584

© 2023 Ji M et al. *JAMA Network Open*.

Race/ethnicity (%)				
African-American	67.2	69.55	60.23	**<0.001**
Caucasian	14.08	12.14	14.81	
Hispanic	14.77	14.68	20.21	
Other	3.94	3.63	4.74	
Body mass index (kg/m^2, mean, SD)	28.75 (7.38)	28.98 (7.59)	29.11 (7.53)	0.154
History of diabetes (%)	8.55	6.98	9.75	**0.028**
History of hypertension (%)	29.27	23.76	25.67	**<0.001**
Cardiac death (%)	31.09	30.01	34.76	**0.006**
KDPI (mean, SD)	0.45 (0.26)	0.40 (0.25)	0.41 (0.25)	**<0.001**

© 2023 Ji M et al. *JAMA Network Open*.
