# Supplementary material for: Patterns in Use and Transplant Outcomes Among Adult Recipients of Kidneys From Deceased Donors With COVID-19
Source: JAMA Netw Open. 2023 May 30;6(5):e2315908. doi: 10.1001/jamanetworkopen.2023.15908 (PMC10230314; doi:10.1001/jamanetworkopen.2023.15908)
Supplement: Supplement 2. — Data Sharing Statement [file jamanetwopen-e2315908-s002.pdf]

Data Sharing Statement

Ji. Patterns in Use and Transplant Outcomes Among Adult Recipients of Kidneys From Deceased Donors With COVID-19. *JAMA Netw Open*. Published May 30, 2023. doi:10.1001/jamanetworkopen.2023.15908

Data
Data available: Yes
Data types: Data (not involving human participants)
How to access data: https://optn.transplant.hrsa.gov/data/
When available: beginning date: 01-01-2013

Supporting Documents
Document types: None

Additional Information
Who can access the data: anyone requesting the data
Types of analyses: for a specified purpose
Mechanisms of data availability: with a signed data access agreement
